# Epidemiological characteristics of deaths from road traffic accidents in Addis Ababa, Ethiopia: a study based on traffic police records (2018–2020)

**DOI:** 10.1186/s12873-023-00791-0

**Published:** 2023-02-20

**Authors:** Micheal Alemayehu, Asfawesen Woldemeskel, Ararso Baru Olani, Tariku Bekelcho

**Affiliations:** 1 Department of Emergency and Critical care, Tirunesh Beijing General Hospital, Addis Ababa, Ethiopia; 2 Department of Medicine, College of health sciences, Ethiopia police University, Addis Ababa, Ethiopia; 3 Department of Nursing, College of health sciences, Arba Minch University, Arba Minch, Ethiopia

**Keywords:** Epidemiological characteristics, Death, Road traffic accident

## Abstract

**Introduction:**

Road traffic accidents are a major cause of fatal and nonfatal injuries, causing permanent disabilities, and other indirect health complications. Each year, road traffic accidents (RTA) cause a lot of fatalities and injuries in Ethiopia, putting the country among the list of the most affected countries by RTA in the world. Despite the high rates of road traffic collisions in Ethiopia, very little is known about the factors that contribute to fatal RTA in the country.

**Objectives:**

the objective of this study is to assess the epidemiological characteristics of deaths from road traffic accidents in Addis Ababa, Ethiopia: A study based on traffic police records (2018–2020).

**Method:**

A retrospective observational study design was conducted used in this study. All road traffic accident victims reported to Addis Ababa police station between 2018 and 2020 were study population and the collected data was evaluated with Statistical Package for the Social Sciences (SPSS) version 26 software. Binary logistic regression model was used to indicate the association between dependent and independent variables. Statistically, significant associations were declared at P < 0.05.

**Result:**

There were 8458 recorded road traffic accidents in Addis Ababa during 2018–2020. Among these accidents, 1274 (15.1%) caused death while 7184 (84.1%) caused an injury. Males accounted for 77.1% of the decedents (sex ratio of almost 3.36:1). The majority 1020 (80%) of the fatality occurred on a straight road and 1106 (86.8%) of the fatality occurred in dry weather. Weekday 1.243 (AOR, 1.234, 95 CI, 1.071–1.443), driver educational status below grade twelve 0.326(AOR 0.326, CI, 0.285–0.374), and commercial truck vehicle 1.682 (OR, 1.696, CI, 1.410–2.040) were statistically associated with fatality after adjusting for potential confounding variables.

**Conclusion:**

The prevalence of RTA fatality in Addis Ababa is high. The accidents that occurred during the weekdays were more fatal. Driver’s educational status, weekdays, and vehicle type were factors associated with mortality. There is a need to introduce road safety interventions that targeted identified factors in this study to reduce fatalities attributed to RTIs.

## Introduction

Road traffic injury (RTI) accounts for one of the leading causes of death and disability worldwide [1]. Globally, over 3500 people die every day on the roads, which amounts to nearly 1.3 million preventable death every year as a result of road traffic accidents. In addition, 20 to 50 million more suffer non-fatal injuries, with many of them resulting in disabilities [2].

RTIs are the leading cause of death for young people aged 15–29 years [3]. However, it continues to be a neglected issue in many developing countries, and the health sector has been slow to recognize RTIs as a priority for public health [4]. Numerous studies indicate that RTIs can be easily prevented and many high-income countries have done so by implementing proven and cost-effective interventions [5].

RTI is owing to increase in developing countries as the number of vehicle owners is increasing. Nearly 90% of all road fatalities occur in low- and middle-income countries, which own only 48% of the world’s registered vehicles. In Low- and middle-income countries (LMICs), road traffic costs are estimated at $100 billion every year, or 1–3% of the gross national product, because of disability, premature death, loss of productivity, medical expenses, and material damage [6].

The burden of RTI is higher in Africa due to the high number of road users who are exposed, overcrowding, poor transportation conditions such as lack of seat belts, and hazardous driving conditions. Furthermore, the under-reporting has masked the impact of the problems in Africa [7].

Identifying factors associated with fatal crashes is one of the most efficient and effective ways to take the right decision for improving road safety and reducing crashes [8]. Diverse studies have been carried out worldwide and identified that most of the underlying factors contributing to fatal road crashes are attributable to human factors, and vehicular and environmental factors [9]. However, factors contributing to crashes could vary across the country as well as across the different roadways within the country.

Each year, road traffic accidents cause a lot of fatalities and injuries in Ethiopia, making it one of the worst countries in the world for these sorts of accidents. Thousands of people are killed each year, and the majority are in the economically active population. WHO report states that the prevalence of RTIs was 25.3 per 100,000 population in Ethiopia in 2013, which is one of the highest rates worldwide [10]. Despite the high rates of road traffic collisions in Ethiopia very little is known about the factors that contribute to fatal road traffic accidents in the country.

This study aimed to examine the Epidemiological characteristics of deaths from road traffic accidents among victims reported to Addis Ababa town police stations, based on the secondary data of a traffic police report between 2018 and 2020, Ethiopia. We hope, the findings of the study will help to bring forward the agenda of road safety to the attention of public health decision-makers. Further, the study will contribute to the evaluation and monitoring of ongoing interventions in the country.

## Methods

### Study area

The study was conducted in Addis Ababa, which is the capital city of Ethiopia, and the seat of the African Union. The city is divided into 11 sub-cities and 116 weredas (Amharic equivalent for districts). Currently, the city has a population of 4.8 million people in the urban area and 2.7 million people in its city area. As a fast-growing city, Addis Ababa exhibits high levels of social, economic, and structural changes. The majority(60%) of registered vehicles in Ethiopia are found in Addis Ababa [11].

### Study design and study period

A retrospective cross-sectional observational study design was carried out to assess the epidemiological characteristics of deaths from road traffic accidents in Addis Ababa, Ethiopia: using data registered by traffic police in Addis Ababa between 2018 and 2020.

### Sampling approach

Since the study used readily available data, no sample size calculation was carried out in advance.

### Data collection

Secondary data were extracted from Addis Ababa traffic police records by using a data extraction tool developed from the traffic police registry format. Variables such as socio-demographic characteristics of victims and drivers (age, sex, education status, driving experience), vehicle-related factors (vehicle ownership), and environmental variables (lighting conditions, road types, and weather conditions) were gathered.

### Data entry, processing, and analysis

Data were checked for completeness and entered into EPI data version 4.6 for validation. Then, exported to SPSS version 26 for analysis. Descriptive statistics such as frequency and percentage were used to summarize the data while tables and graphs were used for the presentation of the data. Purposive selections of the variable with a p-value of < 0.25 on bivariate analysis were considered to identify factors associated with outcomes of RTA. A binary logistic regression model was fitted to control the possible effect of confounders. Finally, the variables which have an independent association with fatality were identified and reported with OR, with 95%CI and a p-value less than 0.05. Model fitness was also checked by using the Hosmer-Lemeshow test.

### Ethical consideration

The ethical clearance was obtained from Ethiopia Police University. Informed consent was obtained from the Addis Ababa traffic police commission before proceeding with data collection from charts. Informed patient consent was not obtained since the waiver for patient informed consent was obtained from an institutional review board of the Ethiopian Police University because of the retrospective nature of data collection. Anonymity was maintained by not encoding any personal information, and only serial numbers were assigned to the injured individuals. This study also complies with the Declaration of Helsinki.

## Result

### Socio-demographic characteristics of road traffic Injury victims and drivers

There were 8458 recorded RTIs in Addis Ababa during 2018–2020. Among these injuries, 1274 (15.1%) caused death while 7184 (84.1%) caused an injury. The majority 7307 (86.4%) of the driver involved in the Injury were male. The mean age of the drivers was 33 (± 10). Males accounted for 77.1% of the decedents (sex ratio of almost 3.36:1) and the mean age of the victims was 33 (± 21). The majority of drivers 5268(63.1%) were below grade 12. (Table 1)

**Table 1:** Socio-demographic characteristics of Victims and drivers involved in fatal and nonfatal road traffic injuries reported to police stations from 2018 to 2020, Addis Ababa Town, Ethiopia

Variables	Category	Frequency of fatalities of RTAs			
		Nonfatal		Fatal	
		N	%	N	%
Age of driver involved in fatal or nonfatal	< 18	106	1.5	20	1.56
	18–30	3316	46.2	577	45.3
	31–50	3276	45.6	592	46.46
	> 50	486	6.7	85	6.68
Age of the victim	< 18	1794	25	102	8
	18–30	2282	31.76	398	31.2
	31–50	1726	24	503	39.5
	> 50	1382	1924	271	21.3
Vehicle ownership	Private	5921	82.42	994	78
	Governmental	788	11	201	15.78
	Others	475	6.58	79	6.22
Drivers’ educational status	Below Grade 12	4802	66.84	466	63.4
	Grade 12 and above	2382	33.16	808	36.6

### Characteristics of RTIs according to time, place, weather conditions, and vehicles type

4897(68.16%) of the injury and 1020 (80%) of the fatality occurred on the straight road. The vehicle used for public transportation caused the majority of the injury and the majority of the fatality was caused by commercial truck vehicle 487(38.2%). More than three forth 6354(88.44%) of the injury and 1106 (86.8%) of the fatality occurred during dry weather. (Table 2)

**Table 2:** Characteristics of RTA’s Fatal according to time, place, Weather condition, and Type of Vehicles Involved in Accidents from 2018 to 2020, Addis Ababa Town, Ethiopia

Variables	Category	Frequency of fatalities of RTAs			
		Nonfatal		Fatal	
		N	%	N	%
Time at which the accident occurred	6:00 AM-12:00 PM	839	11.7	211	14.7
	12:00 PM-6:00 PM	2212	30.8	335	26.9
	6:00PM-12:00AM	2090	29.1	262	21
	12:00AM-6:00AM	2043	28.4	466	37.4
Season	Autumn	1925	26.8	321	25.2
	Winter	1674	23.3	341	26.77
	Spring	1647	22.9	295	23.15
	Summer	1938	27	317	24.88
Whether Condition	Dry	6354	88.44	1106	86.8
	Foggy/cloudy	347	4.8	93	7.3
	Rainy	477	6.76	65	5.9
Types of vehicles involved in the accident	Two or three-wheeler	1271	17.7	221	17.3
	Public transport	2275	31.67	333	26.1
	Commercial truck	2016	28	487	38.2
	Automobile	1622	22.63	233	18.4
Road types	Straight	4897	68.16	1020	80
	Curved	1838	25.58	87	6.8
	Tilted	449	6.26	167	13.2

### Victims of the accidents

**Fig. 1:**
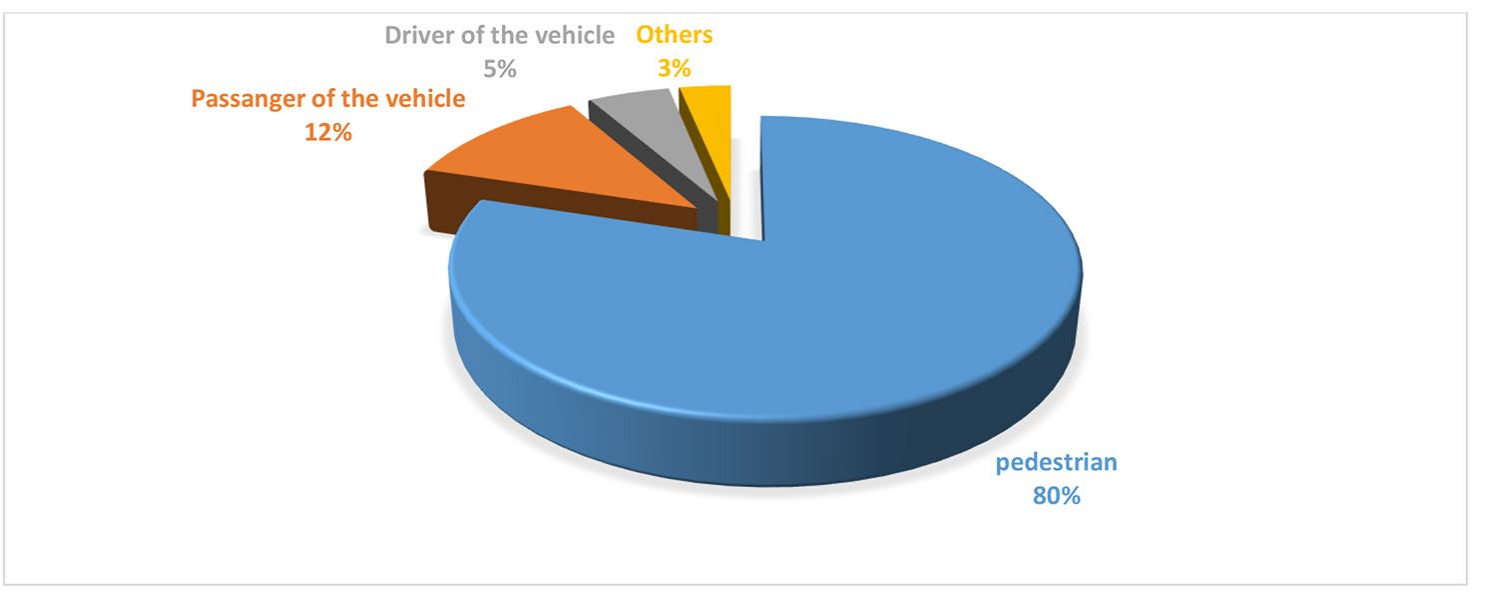
Fatality of Victims Involved in Accidents from 2018 to 2020, Addis Ababa Town, Ethiopia

More than three-fourths of 1017 (79.8%) of the fatality have occurred in pedestrians followed by the passenger of vehicle 151(11.85%) (Fig. 1*)*.

### Fatality pattern in days

**Fig. 2:**
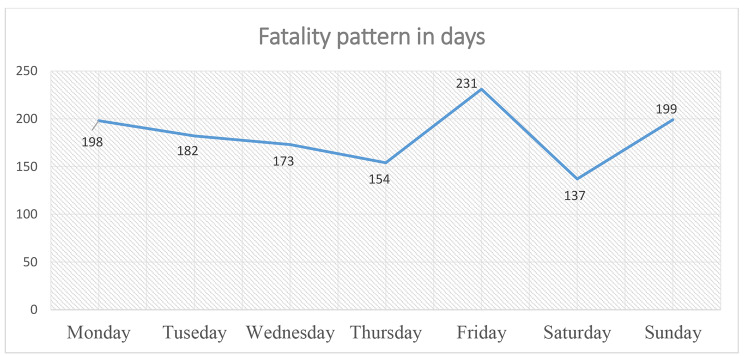
Fatality pattern in days from 2018 to 2020, in Addis Ababa Town, Ethiopia

Regarding the fatality pattern by days, most of the fatalities (231) occurred on Friday and the least of the recorded fatalities happened on Thursday *(*Fig. 2*).*

### Factors associated with deaths from RTAs

In bivariate regression analysis season, days, light condition, driver educational status, vehicle type, and vehicle owner were statistically associated with fatality at a p-value of less than 0.25.

In multivariable regression analysis day, driver educational status, and vehicle types were statistically associated with road traffic accident deaths after adjusting for potential confounders.

The fatal RTAs during a weekday were higher by 1.243 than the fatality during the weekend (P-value = 0.004 ). Drivers who had an educational level below grade 12 caused higher mortality by 32.6% than the drivers who had educational status above grade 12 (P-value = 0.000 ). The fatality caused by commercial trucks was higher by 1.682 times than the fatality caused by automobile vehicles (P-value = 0.000 ) (Table 3).

**Table 3:** Logistic regression analysis of factors associated with fatality in road traffic accidents reported to police stations from 2018 to 2020, Addis Ababa Town, Ethiopia

Variables		Outcome		COR (95%CI)	AOR (95%CI)	P Value
		Fatal	Non Fatal			
Season	Autumn	321	1925	1.019(0.862–1.205)	1.002(0.832–1.206)	0.983
	Winter	341	1674	1.245(1.055–1.471)	1.100 (0.919–1.335)	0.283
	Spring	295	1647	1.095(0.922-1.300)	1.092 (0.906–1.316)	0.354
	Summer	317	1938	1	1	
Day	Weekday	937	4982	1.229(1.075–1.405)	1.243 (1.071–1.443)	0.004
	Weekend	337	2202	1	1	
Light situation	Daylight	728	4133	1.834(1.490–2.257)	0.911 (0.797–1.043)	0.177
	Night	546	3051	1	1	
Driver’s educational level	Below Grade 12	808	2382	0.286(0.253–0.324)	0.326 (0.285–0.374)	0.000
	Grade 12 and above	466	4802	1	1	
Vehicle type	Two or three-wheeler	221	1271	1.196(0.955–1.497)	1.196(0.955–1.449)	0.119
	Public transport	333	2275	1.019(0.852–1.219)	1.260(1.041–1.526)	0.018
	Commercial truck	487	2016	1.682(1.420–1.992)	1.696(1.410–2.040)	0.000
	Automobile	233	1622	1	1	
Vehicle owner	Private	1104	6246	1	1	
	Governmental	101	588	1.210(0.895–1.397)	0.840 (0.6370–1.109)	0.218
	Others	69	350	1.284(0.982–1.677)	0.957 (0.679–1.350)	0.802

## Discussion

This study based on traffic police records data indicated that the rates of fatalities secondary to RTAs are high in Addis Ababa and more than three-fourths of the fatality have occurred in pedestrians. Further, fatalities are more frequent among males and adults 31–50 years of age. The majority of the fatality was caused by a commercial truck vehicle.

The prevalence of mortality in this study was 15.1%, this finding is higher than the study conducted in Turkey, Libya, and Guinan which is 0.3%,7%, and 1.4% respectively [12–14]. However, The study conducted in Italy, Nigeria, and Ethiopia showed a higher mortality rate which is 37%,23.7%, and 25% respectively. This difference might be due to the study design, sample size, and the lockdown during the COVID-19 era.

According to this study, the majority of 5747 (67.9%) victim was male, and similarly, the mortality was higher 966(77.1%) in the male sex. This finding is similar to the report of WHO which indicates that more than 75% of road traffic deaths happened in males. This could be due to the greater exposure to the traffic of the males compared to females as drivers or riders.

More than 73.5% of fatal RTA injuries occurred on weekdays. From the multivariate analysis, the chance of the occurrence of fatal RTA injuries on weekdays was 1.243 times greater as compared with weekends. This study is inconsistent with other reports [15, 16, 18]. The possible reasons are due to people in Addis Ababa town traveling more on the weekdays, as people typically went to work or school and move around for business issues on the weekdays.

Even though, drivers’ educational level plays a crucial role in a road traffic accident. In this study, those who have an educational background above grade 12 caused the majority of the fatality. In addition, those who were below grade twelve were less likely to cause fatality as compared to those who were above grade 12. This finding is contradicted by various studies which show lower educational status is the factor contributing to fatality [15, 19, 20, 21]. This variation might be due to the study setting.

In this study, most of the fatality reported in the winter season. Winter is a dry and hottest season in Ethiopia and during dry and hot time RTAs fatality were likely to increase which was consistent with another study [22, 23, 24]. Our findings are different from a study conducted in Iran and Ethiopia which shows the majority of the fatality reported in the rainy season [15, 24]. The variation might be attributed to the majority of outdoor activities being more common in the dry season.

In this study majority of 487 (5.76%) the fatality was due to commercial trucks followed by public transport 333(3.94%). This finding is in line with the report by Tekepa et al. which showed a majority of the mortality were due to commercial truck [25]. However, the study conducted in Finote Selam and Burayu revealed that most of the mortality was due to public transportation. This discrepancy might be due to the geographical location and the socioeconomic activity difference between the cities [15, 19]. This study also revealed commercial trucks were significantly associated with mortality.

Conversely, When interpreting the study’s findings, it is important to consider the following limitations. Despite the large sample size we used, Records of road traffic accidents from the Addis Ababa Traffic Police commission provide a single reason for the accidents mainly focusing on drivers but the causes of road traffic accidents are multi-factorial. The study also only assessed the socio-demographic differentials of fatality and did not explore more critical risky behaviors of drivers.

## Conclusion

The prevalence of road traffic accident fatality in Addis Ababa is high. Mortality more commonly occurs during the weekdays and winter seasons when people are more active and participate in outdoor activities frequently. Driver educational status, weekdays, and vehicle type were factors associated with mortality. There is a need to introduce road safety interventions that targeted identified factors in this study to reduce fatalities attributed to RTA. For effective interventions, further prospective and qualitative studies are needed to analyze crash characteristics as well as driver behavior and perception.

## Data Availability

Data generated from this study on which the results are based are available from the corresponding author on reasonable request.

## Abbreviations

AB: Ararso Baru
AW: Asfawesen Woldemeskel
LMIC: Low- and middle-income country
MA: Micheal Alemayehu
PI: Principal investigator
RTA: Road traffic accident
RTC: Road traffic collision
SD: Standard deviation
SPSS: Statistical package for social sciences
TB: Tariku Bekelcho
WHO: World health organization

## References

[1] World Health Organization. 2009., *Global status report on road safety: time for action*. Geneva, Switzerland: World Health Organization, 2009.

[2] WHO., “World report on road traffic injury prevention,” 2019.

[3] World Health Organization and World Bank (2013). The world report on road traffic injury prevention.

[4] Watkins K. “Safe and sustainable roads: An agenda for Rio + 20,” 2012. [Online]. Available: http://www.youthforroadsafety.org/uploads/nieuws_bijlagen/rio_20_report_lr.pdf

[5] Mohan D. Road safety in less-motorized environments: future concerns. Int J Epidemiol. Jun. 2002;31(3):527–32. 10.1093/ije/31.3.527. 10.1093/ije/31.3.527 12055145

[6] WHO., “Global Status Report on Road Safety,” 2013.

[7] Lagarde E. Road Traffic Injury is an escalating Burden in Africa and deserves Proportionate Research efforts. PLoS Med. Jun. 2007;4(6):170. 10.1371/journal.pmed.0040170. 10.1371/journal.pmed.0040170 PMC1896192 17593893

[8] Howe LD, Huttly SRA, Abramsky T. “Risk factors for injuries in young children in four developing countries: the Young Lives Study,” *Trop Med Int Health*, vol. 11, no. 10, pp. 1557–1566, Oct. 2006, doi: 10.1111/j.1365-3156.2006.01708.x. 10.1111/j.1365-3156.2006.01708.x 17002730

[9] Adem HA, Demena M, Asefa F, Gobena T. “Road Traffic Accidents Fatality and Associated Factors in Southwest Shoa, Central Ethiopia.” East African Journal of Health and Biomedical Sciences, Jan. 2020. [Online]. Available: https://www.researchgate.net/publication/341431631

[10] Samson F. “Analysis of Traffic Accident in Addis Ababa. Traffic Simulation.” Addis ababa university; 2006.

[11] Addis Ababa City Roads and, Bureau T.“Ethiopia Transportation System Improvement Project.”2016.

[12] Erenler AK, Gümüş B. “Analysis of Road Traffic Accidents in Turkey between 2013 and 2017,” *Medicina*, vol. 55, no. 10, p. 679, Oct. 2019, doi: 10.3390/medicina55100679. 10.3390/medicina55100679 PMC6843299 31600894

[13] Bodalal Z, Bendardaf R, Ambarek M. A study of a decade of Road Traffic Accidents in Benghazi - Libya: 2001 to 2010. PLoS ONE. Jul. 2012;7(7):e40454. 10.1371/journal.pone.0040454. 10.1371/journal.pone.0040454 PMC3394723 22792332

[14] Kourouma K, et al. Frequency, characteristics and hospital outcomes of road traffic accidents and their victims in Guinea: a three-year retrospective study from 2015 to 2017. BMC Public Health. Jul. 2019;19(1):1022. 10.1186/s12889-019-7341-9. 10.1186/s12889-019-7341-9 PMC6668061 31366335

[15] Tadege M. Determinants of fatal car accident risk in Finote Selam town, Northwest Ethiopia. BMC Public Health. Dec. 2020;20(1):624. 10.1186/s12889-020-08760-z. 10.1186/s12889-020-08760-z PMC7201712 32375719

[16] Hingson R, Winter M (2003). Epidemiology and consequences of drinking and driving. Alcohol Res Health.

[17] Mishra B, Sinha N, Sukhla S, Sinha A (2010). Epidemiological study of road traffic accident cases from Western Nepal. Indian J Community Med.

[18] Gray RC, Quddus MA, Evans A. Injury severity analysis of accidents involving young male drivers in Great Britain. J Saf Res. Jan. 2008;39(5):483–95. 10.1016/j.jsr.2008.07.003. 10.1016/j.jsr.2008.07.003 19010122

[19] Hordofa GG, Assegid S, Girma A, Weldemarium TD. “Prevalence of fatality and associated factors of road traffic accidents among victims reported to Burayu town police stations, between 2010 and 2015, Ethiopia,” *Journal of Transport* & *Health*, vol. 10, pp. 186–193, Sep. 2018, doi: 10.1016/j.jth.2018.06.007.

[20] Konlan KD et al. “Prevalence and Pattern of Road Traffic Accidents among Commercial Motorcyclists in the Central Tongu District, Ghana,” *The Scientific World Journal*, vol. 2020, pp. 1–10, Jun. 2020, doi: 10.1155/2020/9493718. 10.1155/2020/9493718 PMC7285403 32565754

[21] Sami A (2013). Educational level and age as contributing factors to road traffic accidents. Chin J Traumatol.

[22] Shahbazi F, Soori H, Khodakarim S, Ghadirzadeh M, Nazari SH (2019). Analysis of mortality rate of road traffic accidents and its trend in 11 years in Iran. Arch Trauma Res.

[23] Algahtany MA, Trend “Secular, Variation S. Epidemiological Pattern, and Outcome of Traumatic Head Injuries Due to Road Traffic Accidents in Aseer, Saudi Arabia,” *IJERPH*, vol. 18, no. 12, p. 6623, Jun. 2021, doi: 10.3390/ijerph18126623. 10.3390/ijerph18126623 PMC8296390 34202974

[24] Majdzadeh R, Khalagi K, Naraghi K, Motevalian A, Eshraghian MR. Determinants of traffic injuries in drivers and motorcyclists involved in an accident. Accid Anal Prev. Jan. 2008;40(1):17–23. 10.1016/j.aap.2007.03.019. 10.1016/j.aap.2007.03.019 18215528

[25] Tékpa BJD, Diemer HC, Issa Mapouka PA, Ndoma Ngatchokpo V, Gassima B, Nali MN. “Mortality during road traffic accidents in Bangui, Central African Republic,” *Médecine et Santé Tropicales*, vol. 27, no. 4, pp. 426–430, Oct. 2017, doi: 10.1684/mst.2017.0745. 10.1684/mst.2017.0745 29313512

